# Fostering healing through mindfulness in the context of medical practice

**DOI:** 10.3747/co.v16i2.413

**Published:** 2009-03

**Authors:** P.L. Dobkin

**Keywords:** Healing, mindfulness, medicine

Suffering is an affective experience of unpleasantness and aversion associated with harm or threat of harm. Suffering may be physical or mental (or both), depending on whether it is linked primarily to the body or the mind. Often it is precipitated by illness, especially when patients feel a threat to personal identity. Patients may experience isolation, a sense of loss of control and predictability in their lives. Mount and colleagues^1^ identified themes revealed by palliative care patients: Those who suffered and faced anguish felt a sense of disconnection from self, others, and the phenomenal world; they had a crisis of meaning with an inability to find solace; they were preoccupied with the future or the past; they maintained a sense of victimization; and they needed to be in control.

Many physicians practicing Western medicine have mastered skills aimed at diagnosing and curing diseases, and yet they may be at a loss when it comes to relieving suffering. With the advent of specialization, physicians have tended to focus on physical data (for example, test results) or on particular systems (cardiovascular, for instance) rather than on the whole person. Even though they acknowledge that psychosocial (and spiritual) factors may influence patients’ outcomes, physicians may have qualms about using that knowledge, perhaps because they consider it to be outside their realm of expertise—or more practically, because they think it too time consuming.

## DISTINGUISHING “CURING” AND “HEALING”

Hutchinson and colleagues 2 distinguish “curing” from “healing”—the former being an action carried out by a health care practitioner to eradicate disease; the latter being a process leading to wholeness and relief of suffering in response to injury or disease. The roles of physicians and patients differ considerably for curing and healing to occur. A physician draws upon expertise concerning disease to bring about a cure (when possible), but must shift positions when healing is the aim.

Healing is a process involving movement toward an experience of integrity and wholeness in response to injury or disease. It depends on an innate potential within a patient 1. Hutchinson *et al*. 2 observe that healing may occur upon acceptance of things as they are, including the fact that change is a constant factor in life. Mount *et al*.^1^ note that acceptance of self and personal situation is not a form of resignation; instead, it is an active integration of reality that frees a person to discern and opt for that which is possible given the constraints of the circumstances. For example, a woman who has been treated successfully for early-stage breast cancer needs to make choices about how to resume activities even though she is anxious about recurrence. By acknowledging and facing her fears (rather than repressing or escaping them), she can strengthen her resolve to live the rest of her life fully.

Egnew 3 conducted a qualitative study that involved an inquiry by Drs. Cassell, Hammerschlag, Inui, Kubler–Ross, Saunders, Siegel, and Stephens about the meaning of healing. A distillation of the interview data led to the statement “Healing is the personal experience of the transcendence of suffering” (p. 258). These well-respected allopathic physicians agreed that the healing process takes place within a trusting relationship. This assertion is consistent with the qualitative data reported by Hsu *et al*.^4^ , who conducted, with patients, physicians, and other health care professionals, focus groups pertaining to healing. A consensus that healing is both a personal and an interpersonal experience emerged. Emphasis was placed on communication, information sharing, support, empathy, and compassion. For instance, when a relapse occurs, the words spoken by the physician, the tone of voice used, the manner in which the patient is invited to integrate undesired news, the ability of both parties to explore their respective reactions, and the respect shown for the patient’s preferences and needs will influence the healing process.

Kearney 5 posits that providing a safe place in which patients can regain a sense of integrity and wholeness is part of the health care mandate. This place is more than a hospital corridor or an examining room; it encompasses the space in which expressions of doubts, dread, and hope can be heard. Mount 6 emphasizes the importance of inviting a meaningful exchange between two equal individuals, one who happens to be a doctor, and the other, a patient. For example, by being present to and accepting personal sorrow when communicating bad news about recurrence, the physician (sometimes called the “wounded healer”^7^ ) may be able to contain the patient’s grief.

Because suffering is magnified by a personal perception of being separate and alone, suffering may be alleviated by the presence of another who is able to be with and to bear the distress. A physician can be one such person. The physician may acknowledge the patient’s suffering verbally or otherwise, and may encourage the patient to deal with that which perpetuates it. Fricchione^8^ refers to this situation as the physician’s willingness to provide care by stepping into the “intermediate area” between separation and attachment.

## HOW MIGHT MINDFULNESS REDUCE SUFFERING AND FOSTER HEALING?

Brown and Ryan^9^ consider mindfulness to be an attribute of consciousness; they propose that consciousness encompasses both awareness and attention. When purposefully cultivated, mindfulness results in heightened awareness of inner and outer experiences through focused attention in the present moment.

In the late 1990s, Epstein 10 published an article in *JAMA* titled “Mindful Practice.” That article elaborates on how mindfulness can be brought into the clinical encounter. Epstein says, “Mindful practitioners attend in a nonjudgmental way to their own physical and mental processes during ordinary, everyday tasks” (p. 833). By taking this stance, the physician can be open to the whole person who presents as a patient and can skilfully treat that patient. According to Epstein, the goal of mindfulness is informed compassionate action incorporating relevant information, making correct decisions, and empathizing with the patient as a means of relieving suffering.

In line with the importance of relating to patients in this manner, Stewart^11^ showed the link between effective physician–patient communication and patient health outcomes (that is, emotional health, symptom resolution, functional status, and pain control). He maintained that, for optimal communication to occur, physicians must be “mindful” of themselves, the patient, and the context.

## CAN MINDFULNESS BE LEARNED?

Epstein answered the question of whether mindfulness can be learned in the affirmative. Mindfulness is characterized by learned mental habits: attentive observation of self, patient, and context; critical curiosity; beginner’s mind (that is, viewing the situation free of preconceptions); and presence. Presence is defined as “connection between the knower and the known, undistracted attention on the task and the person, and compassion based on insight rather than sympathy” 12.

Epstein proposed an eight-fold method for teaching mindful medical practice^13^: priming, availability, asking reflective questions, active engagement, modeling while “thinking out loud,” practice, praxis (consolidation of knowing through experience), and assessment and confirmation. The method can be integrated directly into medical training by a mentor who also engages in the relevant mental habits when working with patients.

It is recommended that mindfulness be introduced early in medical education^14^ given that Shapiro and colleagues^15^ found that the level of empathy significantly declined in medical students during the period between entry into medical school and the end of the first year. To counter this trend of decline, a program titled “Mindfulness-based Stress Reduction” has been provided, with positive results, to medical students and physicians in various medical schools around the world. In a randomized clinical trial for health care professionals, Shapiro *et al*.^16^ found that following the program, participants reported reduced stress levels, increased quality of life, and more self-compassion. In a study with a larger sample size of medical students, Rosenzweig and colleagues^17^ reported similar results.

Being a physician is both a privilege and responsibility. Mindfulness enhances the physician’s ability to bring awareness to the treatment of another human being^18^ . It is not what is done, but how it is done that matters most. It is not how much time is spent with a patient, but rather what transpires within that time. Physicians need to be as comfortable “being” as “doing”—that is, being fully present to the patient and to their own internal processes.

What might this “full presence” look like in the context of a medical encounter?

The physician would be an effective communicator, who listens actively, provides emotional support, relates with compassion, and is flexible. The physician would encourage the patient to explore the meaning of illness and to grow from the experience, no matter the physical condition or prognosis^3^ . The physician would be committed to the patient, offering generosity and patience. The importance of continuity of care would be recognized and acted upon^18^ .

## CONCLUSIONS

To practice medicine in this way—that is, to cure when possible and to foster healing even in the absence of cure—the physician needs to add the form of consciousness called mindfulness to the traditional “black bag.” This state of consciousness can be taught and learned through practice. Numerous medical schools around the world have recognized the need to broaden training such that curing and caring are equally valued and simultaneously provided in the best interest of the patient. Outcomes may depend upon it.

## CONFLICT OF INTEREST STATEMENT

The author declares an absence of conflicts of interest.
